# Research progress on ferroptosis in the pathogenesis and treatment of neurodegenerative diseases

**DOI:** 10.3389/fncel.2024.1359453

**Published:** 2024-03-07

**Authors:** Lijuan Wang, Xiansong Fang, Baodian Ling, Fangsheng Wang, Yu Xia, Wenjuan Zhang, Tianyu Zhong, Xiaoling Wang

**Affiliations:** ^1^The First School of Clinical Medicine, Gannan Medical University, Ganzhou, China; ^2^Laboratory Medicine, First Affiliated Hospital of Gannan Medical University, Ganzhou, China; ^3^Department of Blood Transfusion, The First Affiliated Hospital of Gannan Medical University, Ganzhou, China

**Keywords:** Alzheimer's disease, Parkinson's disease, amyotrophic lateral sclerosis, Huntington's disease, ferroptosis, pathological mechanism, neurodegenerative diseases, ROS

## Abstract

Globally, millions of individuals are impacted by neurodegenerative disorders including Huntington's disease (HD), amyotrophic lateral sclerosis (ALS), Parkinson's disease (PD), and Alzheimer's disease (AD). Although a great deal of energy and financial resources have been invested in disease-related research, breakthroughs in therapeutic approaches remain elusive. The breakdown of cells usually happens together with the onset of neurodegenerative diseases. However, the mechanism that triggers neuronal loss is unknown. Lipid peroxidation, which is iron-dependent, causes a specific type of cell death called ferroptosis, and there is evidence its involvement in the pathogenic cascade of neurodegenerative diseases. However, the specific mechanisms are still not well known. The present article highlights the basic processes that underlie ferroptosis and the corresponding signaling networks. Furthermore, it provides an overview and discussion of current research on the role of ferroptosis across a variety of neurodegenerative conditions.

## 1 Introduction

Neurodegenerative disorders are a collection of disorders that manifest as a slow degeneration of nerve cells and are particularly common in the elderly. With their insidious onset and irreversible progression, human health is greatly threatened by neurodegenerative diseases. The world population is continuing to age, and as a result, mortality and morbidity rates related to neurodegenerative disorders are rising, significantly raising the socio-economic burden (Zhang et al., 2021). However, these diseases can only be alleviated, and it is difficult to make a breakthrough in their treatment (Heemels, 2016).

Targeting RAS, eratin is a tiny molecule that is selectively deadly and was discovered in tumor cells in 2003. This chemical was shown to induce a distinct kind of non-apoptotic cell destruction (Dolma et al., 2003). An innovative type of iron-dependent cell death termed ferroptosis, which is induced by erastin, was identified in 2012 by Stockwell's laboratory. Ferroptosis differs from autophagy, necrosis, and other types of cellular destruction due to inherited, biochemical, and structural variations (Dixon et al., 2012). The basic ferroptosis process is the Fenton reaction, which is driven by ferrous ions and is responsible for initiating the peroxidation of lipids on the cell surface. This process is defined by diminished mitochondrial cristae, rising membrane density, and atrophy of the mitochondria (Vitalakumar et al., 2021). Considering its particular role in numerous clinical conditions, including brain diseases, tumors, and tissue damage resulting from ischemia-reperfusion, it has garnered significant attention (Conrad et al., 2016; Stockwell, 2022). A substantial body of evidence indicates that ferroptosis contributes to the pathophysiology of neurological disorders, and is associated with peroxidation of lipids, brain iron accumulation, and glutathione deficiency. Such conditions have the potential to trigger a series of events that include neurotransmitter oxidative stress, myelin deterioration, inflammatory activation, loss of neuronal interaction, memory loss, and cellular death. Nevertheless, the mechanism that triggers cell death is unknown. Thus, more research is needed to understand how pathologic traits, pathogenesis, and ferroptosis interact in neurodegenerative diseases from a therapeutic perspective.

## 2 Ferroptosis's relation to other cellular death programs

The Nomenclature Committee on Cell Death divided controlled apoptosis into multiple categories in 2018: pyroptosis, autophagy, ferroptosis, necrosis, and apoptosis. This classification was based on the structural, biochemical, and therapeutic perspectives (Galluzzi et al., 2018). *In vivo*, cell membrane breakdown from oxidative damage causes an overabundance of reactive oxygen species (ROS) that causes lipids to peroxide, which leads to multiple kinds of cellular death (Su et al., 2019). The peroxidation of lipid products interacts with inhibitors and transcription variables, membrane receptors, and initiates apoptotic transmission (Elkin et al., 2018). Since ferroptosis occurs later in membrane rupture, phosphatidylserine ectopics may occur, which is similar to apoptosis (Tong et al., 2022). As a defense mechanism, autophagy divides misfolded proteins and damaged organelles using a dual membrane structure named an autophagosome, then integrates them with lysosomal phagocytosis of contents (Sevenich, 2018). Thus, to preserve equilibrium and regular activity of cells *in vivo*, the autophagic lysosomal pathway is significant. The ferroptosis inducer erastin induces autophagy, and a significant moderator of ferritin destruction and transferrin receptor 1 (TfR1) expression is reactive oxygen species (ROS)-induced autophagy (Park and Chung, 2019). Nuclear receptor coactivator 4 (NCOA4) was shown in research to activate ferritin phagocytosis and heat-shock protein 90 (HSP90)-associated chaperonin-mediated autophagy to promote ferroptosis (Zhou et al., 2020). An inflammatory response can be triggered by pyroptosis, a pro-inflammatory kind of programmable cell death (Chang et al., 2020), and exhibits an acute disruption of the cell membrane and an intracellular secretion of pro-inflammatory molecules, involving interleukin (IL) 18 and IL-1β (Man et al., 2017). It was found that CD8+ T lymphocytes, known as antitumor immune cells, may induce and increase ferroptosis and pyroptosis (Tang et al., 2020). Firstly, CD8+ T-cells may release human granzyme family members (GZMA proteins) to act as cleaving enzymes for Gasdermin- b, Gasdermin structural domain protein family member (GSDMB). Cleavage by GSDMB leads to pyroptosis. However, the Solute carrier family 7 (SLC7A11) member is reduced as a result of CD8+ T cells releasing IFN-γ, causing lipid ROS accumulation and suppressing ferroptosis. Therefore, the combined study of ferroptosis and Pyroptosis promotes in development of cancer therapy. Research showed that cellular damage, such as ferroptosis and pyroptosis, might intensify the process of tau hyperphosphorylation and an inflammatory response linked to AD progression (Qiu et al., 2023). Thus, different cell death processes may interact with each other and be closely linked to disease progression.

## 3 Mechanisms of ferroptosis

Ferroptosis is characterized by unchecked peroxidation of lipids and consequent disruption to the cellular membrane, its onset and development are controlled by intricate signaling and regulatory mechanisms. Susceptibility to ferroptosis depends on iron accumulation, glutathione (GSH) depletion, antioxidant inactivation, polyunsaturated fatty acids (PUFAs), and selenium availability (Jiang et al., 2015; Doll et al., 2017). These proteins or metals are important in regulating the degree of oxidative stress within cells. Iron is a significant trace element involved in multiple physiological processes, such as metabolizing energy, transport of oxygen, and oxygen preservation. The internal balance of iron is carefully controlled by the body. Since iron cannot be removed from the body through clearance mechanisms, its regulation in the body is critical. Irreversible oxidative damage occurs when iron is overloaded and exceeds its storage capacity. Several ROS sources that can trigger ferroptosis include the iron-mediated Fenton reaction, mitochondrial ROS, and membrane-associated ROS carried on by the nitrogen oxides (NOX) family of proteins. Pathological states, in which ROS levels are drastically elevated, can cause severe damage to cellular structures. Ferroptosis is directly mediated by lipid peroxidation. Phospholipids comprising polyunsaturated fatty acids, specifically arachidonic acid (AA) or adrenoic acid (AdA) within the phosphatidylethanolamine (PE) molecule, are the primary peroxidation of lipid precursors in iron overload (Galaris et al., 2019; Zhong et al., 2019; Sun et al., 2020). Particularly, acetyl coenzyme Arachidonic acid lipoxygenase (ALOX), cytochrome P450 oxidoreductase (CPY450), lysophosphatidylcholine acyltransferase 3 (LPCAT3), and synthetase long-chain family 4 (ACSL4) contribute in the favorable control of ferroptosis. This is improved by certain modulations of the autophagic degradation pathway, causing peroxidation of lipids and promote iron deposits. In conclusion, ferroptosis is a type of lipid peroxidation that is dependent on iron and ultimately causes a disruption in the structure of the cell membrane, resulting apoptosis.

### 3.1 Iron

Nearly all organisms contain iron, an important trace metal that contributes to various biological processes including metabolizing energy, nucleic acid production, and regeneration. Intracellular iron equilibrium, which is attained by an exacting control on absorption, transport, storage, and release mechanisms, is necessary to maintain the precise metabolism of iron. The small intestine's duodenum and upper jejunum are where iron ions are mainly absorbed. After escaping by intestinal luminal capillaries' endothelial cells, iron ions are attached to transferrin (TRF) in the plasma. They then attach to transferrin receptor 1 (TFR1) and are taken up by lattice protein endocytosis (Koleini et al., 2021). After iron uptake, it is reduced from trivalent to divalent iron in acidic endosomes via prostate hexagonal transmembrane epithelial antigen 3 (STEAP3) and discharged by divalent metal transporter protein 1 (DMT1) into the cell's cytoplasm for cellular usage (Luck and Mason, 2012). Unstable iron pools within cells can become saturated with excessive iron. Such pools of free divalent iron have the potential to induce ROS, particularly hydroxyl radicals, which are produced by the Fenton response. Polyunsaturated membrane lipids are damaged by deposited ROS, which impedes cellular activity and leads to cell destruction. When available iron exceeds biological requirements, cytoplasmic ferritin converts divalent iron into trivalent iron and stores it in ferritin complexes. Thus, ferritin abundance controls ferroptosis sensitivity: ferritin raises the susceptibility to ferroptosis when unstable iron pools are limited because it promotes iron ion preservation. In contrast, when ferritin is reduced, iron escapes into the unstable iron pool and improves ferroptosis susceptibility. Based on studies, the main mechanism by which iron is liberated from saturated ferritin is nuclear receptor coactivator 4 (NCOA4)-mediated directed autophagy. Iron moves into the cell's cytoplasm when ferritin is broken down in the lysosome, where it is transported by NCOA4 (Mancias et al., 2014; Yambire et al., 2019). By facilitating the release of excess iron into the outside of cells, the iron transporter protein 1 (FPN1) helps to improve *in vivo* homeostasis. High levels of FPN1 reduce iron-mediated ROS (Ward and Kaplan, 2012; Mancias et al., 2014). Ceruloplasmin (CP) also inhibits ferroptosis induced by erastin and GSH peroxidase 4 inhibitors (RSL3) by mediating iron efflux through binding to cell surface FPN1.

Iron-related cell death occurs when iron is overloaded. It's plausible that the oxidative possibility of iron, involving Fenton chemistry, initiates the production of peroxided lipids immediately (Shah et al., 2018). Increased levels of free iron can form unstable iron pools, and an increase in unstable iron pools in cells can cause membrane lipid peroxidation. There are two sources of unstable intracellular iron pools: one is ferritin's specific autophagy by NCOA4 liberates the iron ions from ferritin; the other is heme oxygenase-1 (HO-1), it promotes the breakdown of heme into ferric ions and is controlled with the nuclear factor erythrocyte 2-related factor 2 (NRF2) gene, leading to *ex vivo* and *in vivo* mitochondrial iron overload (Chang et al., 2018). Within the cell, excessive iron produces a high level of free radicals from oxygen, which causes peroxidation of lipids byproducts such as 4-hydroxynonenal (4-HNE), malondialdehyde (MDA), and phospholipid hydroperoxides (PLOOH). These accumulating byproducts have the potential to induce ferroptosis and ultimately damage cells (Liang et al., 2023). Multiple other lab research demonstrate that ferroptosis is triggered when Fe2+ is used as a cofactor for ALOX or CPY450 enzymes, and that unstabilized iron, which does not bind to the enzymes, propagates these peroxides to cause the peroxidation of lipids (Wenzel et al., 2017; Shah et al., 2018). In addition, iron acts as the active center of lipoxygenase, which oxidizes polyunsaturated fatty acids on cell membranes through an iron-ion-dependent chain reaction, thereby promoting the proliferation of phospholipid peroxides and thus causing ferroptosis. Iron in the active center of lipoxygenase exhibits in the formation of toxic lipid peroxides. In brief, iron performs a dual function in ROS production and facilitates the synthesis of ALOX, which elevates lipid peroxide levels during ferroptosis. Under normal physiological conditions, iron autophagy maintains intracellular iron homeostasis. Over-activation of iron autophagy results in excessive intracellular iron deposition, causing glutathione peroxidase 4 (GPX4) levels to fall and GSH to become diminished, which finally causes cell membrane structures to collapse and rupture and iron death. In conclusion, iron excess causes the body's iron regulation to be upset, which results in ferroptosis.

Since iron is important to ferroptosis, the specific iron mechanism has attracted a new wave of attention. Iron-catalyzed lipid peroxidation to generate hydrogen peroxide products, iron-catalyzed cleavage reactions, and truncation of electrophilic reagent oxidation are the three main stages of iron-catalyzed reactions. In addition, other factors involved in iron regulation influence sensitivity to ferroptosis (Patel et al., 2019). Thus, GSH deficiency may promote peroxidation of lipids and provide free Fe2+ for the Fenton reaction, which may damage cell membranes and cause ferroptosis. Based on a new study, prominent protein 2 (PROM2) helps produce multivesicular bodies and exosomes that contain proteins that contain iron, which regulates the level of iron in the body. This mechanism promotes resistance to ferroptosis by facilitating the movement of iron ions outside the cell. Similarly, iron export mediated through FPN1 enhances resistance to iron death. Moreover, glutathione-iron complexes are temporarily formed during the process of absorbing iron. Ferroptosis is inhibited by the iron chaperone poly(rC)-binding protein 1 (PCBP1), which interacts with ferritin and injects Fe2+ into it (Stockwell, 2022).

### 3.2 Lipid peroxidation and ROS

The process by which oxidizing agents, such as non-radical compounds or liberated radicals, target lipids with double bonds between carbons is termed lipid peroxidation. In addition to causing direct harm to phospholipids, this attack acts as a signaling mechanism to initiate a process of cell death. PLOOH is highly biologically active and can cause damage to lipid bilayers of cell membranes (Catalá and Díaz, 2016). Numerous investigations showed that nonenzymatic phospholipid autoxidation or enzymatic phospholipid peroxides ALOX34,38,-40, and CPY450 cause lipid peroxidation (Yang et al., 2016; Wenzel et al., 2017; Chu et al., 2019; Li et al., 2021b). The membrane disruption results from the AA/AdA-PE-OOH formation using lipoxygenase-mediated peroxidation of lipids. Ferroptosis caused by RSL3 and erastin involves enzymes important for the lysophospholipid acylation pathway, including acyl-coenzyme A synthase long-chain family member 4 (ACSL4) and lysophosphatidylcholine acyltransferase 3 (LPCAT3) (Soupene and Kuypers, 2008; Shindou and Shimizu, 2009; Dixon et al., 2015). It found that ferroptosis may be suppressed *in vitro* and *in vivo* by pharmacologically or biologically disrupting the ACSL4-LPCAT3-ALOX cascade. Furthermore, CPY450-mediated lipid peroxidation serves as a substitute for ALOX in ferroptosis, facilitating polyunsaturated fatty acid (PUFA) peroxidation and causing cancerous cell death. Ferroptosis is not usually caused by prostaglandin peroxidase synthase 2 (PTGS2); instead, it is believed to as a biomarker. However, in neuronal cells following brain damage, PTGS2 might mediate ferroptosis. Non-enzymatic pathway products include malondialdehyde (MDA), isoprostane, and 4-HNE. MDA can bind to proteins and DNA to form cross-linked compounds, which can alter membrane structure and function (Zarkovic et al., 2013). Moreover, both AD and PD diseases are pathophysiological associated with increased intracellular MDA (Garcia et al., 2013). The regulation of many transcription variables involving Nuclear factor erythroid 2–related factor 2 (Nrf2) and peroxisome proliferator-activated receptor (PPAR), may stimulate 4-HNE, it can be promoted by various signaling mechanisms, such as those involving caspases and cell cycle controls (Dalleau et al., 2013). Neurodegenerative diseases are exacerbated by imbalanced redox equilibrium, which is brought on by elevated 4-HNE levels (Dalleau et al., 2013). However, the significance of Fenton chemistry and iron-dependent enzymes in initiating the peroxidation of lipids, results in ROS and ultimately causes ferroptosis.

A cascade reaction that occurs inside the organism, oxidative stress (OS) is primarily caused by a disruption in the redox equilibrium and an overabundance of ROS. Among such ROS are peroxides (O_2_·-), hydroxyl radicals (OH·-), peroxyl radicals (RO_2_·-), alkoxyl radicals (RO·-), and certain oxidizing agents/non-radicals, such as hydrogen peroxide (H_2_O_2_), ozone (O_3_), hypochlorous acid (HOCl), and singlet oxygen (1O_2_). The body could be harmed as a result of this exposure. However, the factors that contribute to the production of internal ROS include xanthine oxidase (XO), nicotinamide adenine dinucleotide phosphate (NADPH) oxidase (NOX), and the mitochondrial electron transport chain. Of these, the NOX family which consists of up of five monooxygenases (NOX 1-5) and two dioxygenases (Duox 1-2) produces the majority of ROS in the body (Wingler et al., 2011; Zhang R. N. et al., 2018). Nitrogen oxidase (NOX) produces ROS, including hydroperoxides (Bedard and Krause, 2007). Several studies have shown that a minimum of 3 NOX family members such as NOX1, NOX2, and NOX4 were identified to promote iron-dead cancer cell death (Xie et al., 2017; Yang et al., 2019, 2020). Dipeptidyl peptidase 4 (DPP4) attaches to NOX1 in colon tumors to initiate ROS formation, which encourages ferroptosis (Xie et al., 2017). Additionally, TP53 deletion inhibits the central production of DPP4, which permits membrane-associated DPP4-dependent peroxidation of lipids, ultimately causing ferroptosis (Xie et al., 2017). Mitochondria are the primary intrinsic source of reactive oxygen species (ROS). The electrons that are lost during oxidative phosphorylation in the electron transport chain of the mitochondrial membrane combine with molecules of oxygen to form the anion superoxide (O_2_·-). The voltage-dependent anion channel (VDAC) channel protein on the external mitochondrial membrane is in charge of transferring ions, ATP, and other metabolites into and out of the mitochondria. The main functions of VDAC are to regulate cell metabolism, maintain calcium intracellular homeostasis, and modify apoptotic processes (Skonieczna et al., 2017). Yagoda et al. finds that erastin reacts to VDAC2 effectively, allowing tumor cells to undergo ferroptosis. In the presence of oncogenic RAS, RAS-selective lethal small molecule 5(RSL5) interacts with VDAC 2/3 to generate ROS and activate death mechanisms (Yang and Stockwell, 2008). ROS controls apoptosis, proliferation, and other signaling pathways within cells. However, because of their high reactivity and thus elevated ROS, they react with nearly all kinds of biomolecules (Yagoda et al., 2007). Consequently, many cellular and extracellular components are susceptible to damage by increased and prolonged ROS amounts. For example, proteins and lipids may completely degrade or have permanent functional changes. The oxidization of lipids and proteins can result in genetic modifications and inflammatory reactions, and the development of diseases such as premature aging, functional loss, and neural death. Moreover, protein oxidation may promote the formation of insoluble protein aggregation. Many diseases have their molecular roots in such forms of protein aggregation such as Alzheimer's disease (Qin et al., 2006) and PD (Zhang et al., 2000).

### 3.3 PUFAs

**Figure 1:**
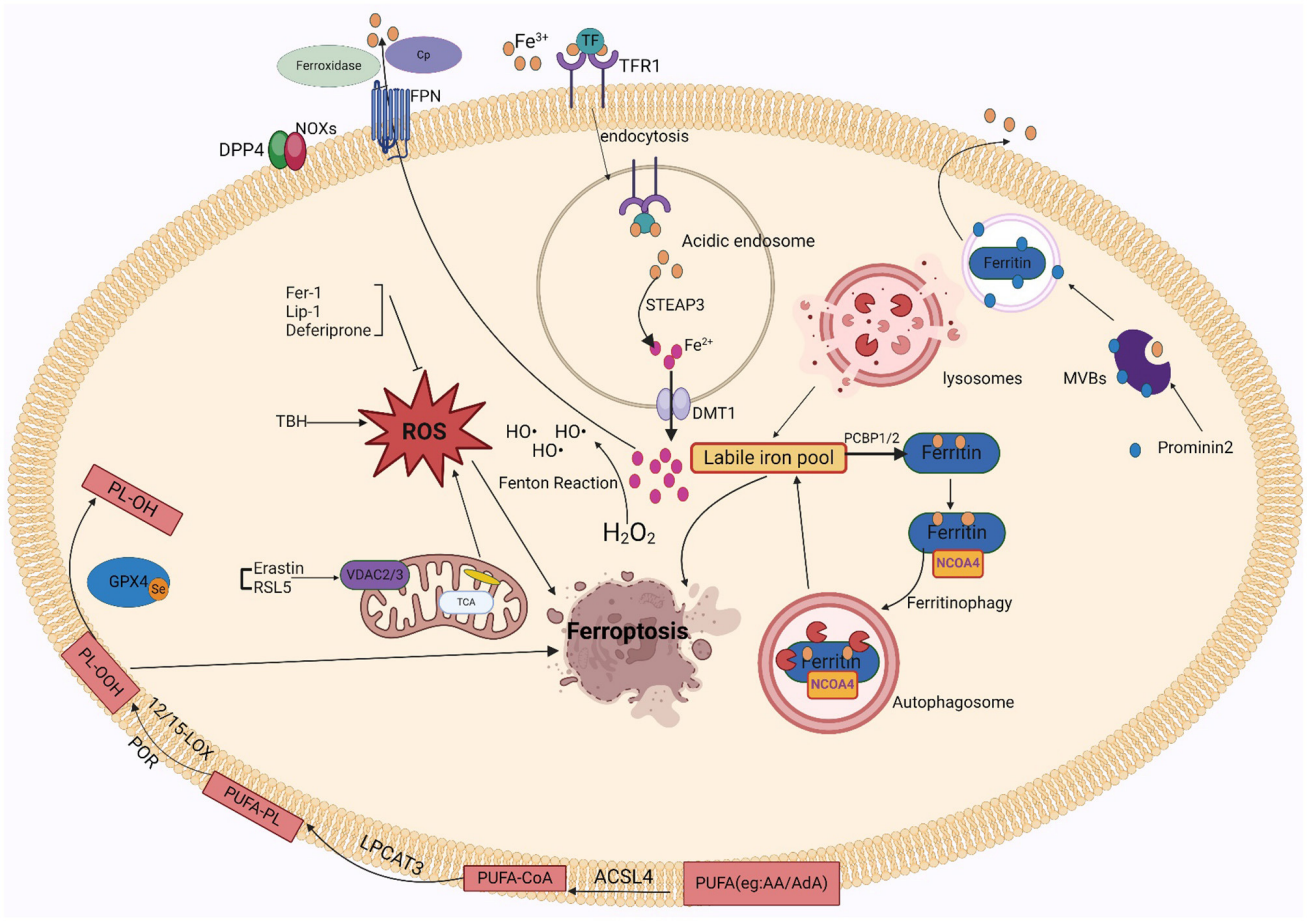
The ferroptosis mechanism involves iron, ROS, and lipid peroxidation.

Fatty acids are processed for multiple purposes, including the synthesis of energy, cell membrane development, and a variety of signaling molecules. They perform a significant part in the cellular metabolism of lipids (Patel et al., 2019). The fatty acids can be divided into three main types: monounsaturated fatty acids (MUFAs, 1 double bond), saturated lipids (no double bonds), and polyunsaturated Lipids (PUFAs, >1 double bond). Under physiological conditions, PUFAs enhance human metabolism and promote fat removal. As components of cell membranes, many biological processes, including as inflammatory processes, immunology, synaptic plasticity, and cell development, are mediated by PUFAs (Guzik et al., 2000). Lipidomics studies have shown that the phospholipids (PLs) of the most vulnerable lipids to peroxidation are PUFAs and cell death. The diallyl carbons (carbon atoms adjacent to two neighboring carbon-carbon double bonds) in PUFA-PLs are chemically susceptible to attack by free radicals, lipoxygenases (LOXs), and ROS, and thus serve as key sites in the lipids that drive ferroptosis (Zorov et al., 2014). Phospholipid-containing PUFAs can form lipid H_2_O_2_ by enzymatic (most notably LOXs) or non-enzymatic (Fenton chemical) oxidation reactions, which bind to iron to produce toxic lipid free radicals that lead to cellular damage. *In vivo*, the most common PUFAs is AA/AdA, which is present in all tissues. The formation of these PUFAs coenzyme A derivatives and their insertion into phospholipids is required for the generation of ferroptosis signals. AA/AdA-PE biosynthesis requires the involvement of ACSL4 and LPCAT3. Thus, deletion of the AA/AdA-PE product depletes the substrate for lipid peroxidation and increases resistance to ferroptosis (Garcia et al., 2013; Dixon et al., 2015; Doll et al., 2017). Cells supplemented with AA or other PUFAs are sensitive to ferroptosis (Yang et al., 2016). Studies have shown that the peroxide site of PUFAs contains deuterium, which can slow the oxidation of PUFAs and inhibit ferroptosis. Deuteration at AA-7 has a protective effect against ferroptosis induced by RSL3 (Gaschler et al., 2018). Thus, the state of PUFAs influences lipid peroxidation. In addition, GPX4 inhibitors can cause hyper oxidation of PUFAs, so the effectiveness and synthesis of PUFAs is a good target for modulating sensitivity to ferroptosis, and modulation of PUFAs may be a good approach for treating diseases associated with ferroptosis. Figure 1 shows the mechanism of ferroptosis. Tables 1, 2 summarize the inducers and inhibitors of ferroptosis.

**Table 1:** Inducers of ferroptosis.

**Inducing agent**	**Action mechanism**	**References**
Erastin	Irreversible inhibition of Xc- uptake of cystine and depletion of GSH levels	Dixon et al., 2012
	Promotion of esterification of AA/AdA-CoA derivatives in PLs, formation of AA/AdA-PE by the action of LPCAT3, and peroxidation	Yuan et al., 2016
	The isoforms of VDAC2 and VDAC3 are involved in erastin-mediated ferroptosis (cancer).	Yagoda et al., 2007
RSL3	Binds and inactivates GPX4 (inhibits GPX4)	Yang et al., 2014; Stockwell, 2022
FIN56	GPX4 breakdown and coenzyme Q10 (CoQ10) depleted activation through the valerate path improve ferroptosis sensitivity	Shimada et al., 2016; Stockwell, 2022
FINO2	Alkoxyl radicals produced by the Fenton reaction with Fe(II) actively help in the peroxidation of lipids. Alternatively, by stimulating LOXs or related iron-dependent enzymes and oxidizing non-heme iron cofactor interaction	Gaschler et al., 2018; Stockwell, 2022
Sorafenib	Lipid ROS accumulation is increased by reducing System XC and blocking cysteine input.	Dixon et al., 2012; Louandre et al., 2013, 2015; Su et al., 2019
Sulfasalazine		
Glutamate		
RSL5	Interacts with VDAC 2/3 to generate ROS	Yang and Stockwell, 2008
Buthionine	Glutamate cysteine ligase (GCL) inhibition causes a lowering of GSH levels.	Yang et al., 2014; Su et al., 2019
L-buthionine sulfoximine (BSO)	Indirect inhibition of GPX4:Inhibition of GSH depletion by GCL	
DPI7 (ML162), DPI10 (ML210), DPI12, DPI13, etc.	Direct inhibition of GPX4	
t-butyl hydroperoxide (TBH)	High levels of ROS and peroxidation of lipids	Wenz et al., 2018

**Table 2:** Inhibitors of ferroptosis.

**Inhibitor**	**Mechanism of action**	**References**
CP	Inhibits ferroptosis brought on by erastin and RSL3 in conjunction with FPN1	Friedmann Angeli et al., 2014; Skouta et al., 2014
Ferrostatin-1(Fer-1)	Suppression of peroxidation of lipids propagation by liberated radical trap	Dixon et al., 2012
Liproxstatin-1(Lip-1)		
CoQ10	Inhibits lipid ROS	
Zileuton	Lowering glutamate-induced oxidative cell death and inhibition of peroxidation of lipids	Liu et al., 2015
Deferiprone	Removes peroxyl radicals and prevents hydrogen peroxide phospholipid synthesis	Liu et al., 2018
Ferroptosis suppressor protein 1 (FSP1)	Iron chelated and delivered to extracellular iron receptors	Kuang et al., 2020
Tetrahydrobiopterin	Antioxidant that reduces the diffusion of fat peroxides	Vasquez-Vivar et al., 2022
N-acetylcysteine	Capture of free radicals	Tardiolo et al., 2018
	Binds ROS	
	Induces GSH production	

## 4 Organelles related to ferroptosis

### 4.1 Lysosomes

It has been shown that the lysosome is an important intracellular storage site for iron ions, which means that it is essential to iron regulation and metabolic activity (Yuan and Ofengeim, 2023). When intracellular levels of iron ions are too high, iron can accumulate in lysosomes, helping in protecting tissues against the negative effects of iron overload (Wang J. et al., 2023). However, ferroptosis may also be triggered when there is too much iron in the lysosome. Second, lysosomes are involved in the release and reuse of iron through their internal acidic environment and abundance of hydrolytic enzymes (Hung et al., 2023). Under specific conditions, such as oxidative stress or cellular injury, the permeability of the lysosomal membrane may increase, causing iron to be released into the cytoplasm from the lysosome. The release of iron may worsen cellular damage and ferroptosis by promoting peroxidation of lipids and the ROS. In addition, as mentioned earlier, lysosomes and autophagy are closely related processes. The mode of intracellular self-digestion identified as autophagy breaks down and recycles intracellular components by encasing intracellular material in autophagic vesicles and fusing with lysosomes (Li et al., 2023). During ferroptosis, autophagy may be activated, resulting in more iron being transported into the lysosome. This further exacerbates iron overload in lysosomes and promotes ferroptosis (An et al., 2023).

Even so, multiple research suggests that by promoting the activity of the antioxidant gene superoxide dismutase 1 (SOD1), activating the nuclear transcription factor EB (TFEB) may prevent lysosome-based ferroptosis. It suggests that there is interaction between the nucleus and the lysosome, which may establish a feedback loop to control ferroptosis (Cai et al., 2024; Calabrese et al., 2024; Shariq et al., 2024). Nevertheless, more research are needed on whether lysosomal cytosolic action leads to membrane remodeling and repair during ferroptosis.

Overall, lysosomes play an integral part in ferroptosis by storing, releasing, reusing, and interacting with processes such as autophagy. Together, these complex processes and mechanisms determine the fate of cells under conditions including iron overload or oxidative damamge. The particular functions and processes that control lysosomes in ferroptosis remain mainly unresolved, necessitating more research and study.

### 4.2 Mitochondria

The main ROS contributor in cells, especially after oxidative phosphorylation is the mitochondria. Localized ROS production not only causes immediate damage to the mitochondria but also modifies the cell's overall oxidative state (Jia et al., 2024a). Research indicates that increased ROS in the mitochondria generates ferroptosis, which can be impeded by antioxidants or enzymes that target the mitochondria (Chen et al., 2024). Secondly, ferroptosis is inhibited partly by certain antioxidant enzymes identified in mitochondria. For example, GPX4 can be identified in the gaps between the cytoplasmic and mitochondrial membranes, and its structure reduces the oxidative stress that destroys mitochondria after a cell dies (Guo et al., 2024).

By living within the matrix of mitochondria, members of the ferromanganese SOD2 may be able to inhibit ferroptosis in non-small cell lung cancer (NSCLC) cells prompted by mitochondrial ROS (Zhao et al., 2023). Moreover, by interacting with ALOX5, microsomal glutathione S-transferase 1 (MGST1) suppresses the ferroptosis and peroxidation of lipids (Zhang et al., 2023).

Moreover, mitochondria control iron metabolism, which affects ferroptosis. The balance of cellular metabolism of iron depends on mitochondria, becoming a significant organelle. By increasing the quantity of iron stored in the mitochondria and lowering cellular ROS levels, overexpression of mitochondrial ferritin can prevent ferroptosis (Xu et al., 2024).

Moreover, the process of apoptosis produced by cysteine deficiency involves mitochondria. The lack of cysteine causes the potential of mitochondrial membranes to become highly polarized and lipid peroxides formation, which finally results in ferroptosis (Du et al., 2024). However, increased polarization of the mitochondrial membrane potential, peroxide-lipid formation, and ferroptosis are lowered when the electron transport chain (ETC) or the mitochondrial tricarboxylic acid cycle (TIC) is inhibited. In conclusion, mitochondria have an important part in ferroptosis through ROS production, the activity of antioxidant enzymes, the control of iron metabolism, and cysteine deprivation. As a result, mitochondria have significance to the ferroptosis process.

### 4.3 ER

The main site for protein production and use in the cell, the Endoplasmic Reticulum (ER), has significance for maintaining cell equilibrium because it contains a significant quantity of membrane lipids. Despite the several stresses, ferroptosis is one to which the ER reacts significantly. Stress in the upper respiratory tract (ER) can set off an unfolded protein process that attempts to restore the proliferative equilibrium. If a cell cannot regain homeostasis, ER stress can lead to cell loss, including ferroptosis (Wang et al., 2024). Second, the peroxidation of lipids in ferroptosis is mainly regulated by the ER. As evidenced by the finding that Fer-1 occurs primarily in the ER, the ER is most important for the peroxidation of lipids during ferroptosis. ER viscosity increases during ferroptosis, possibly due to the aggregation of PUFA-PLs leading to the hardening of the ER (Stockwell, 2022). Ferroptosis is characterized by a substantial anticipation of lipid peroxidation. In addition, significant functions are played by specific ER proteins in controlling ferroptosis susceptibility.

For instance, a transmembrane protein, Stimulator Of Interferon Response CGAMP Interactor 1 (STING1) on the ER translates oxidative responses in the nucleus or mitochondria into a ferroptosis response. STING1 is ligand-activated in response to the oxidized nucleic acid base 8-hydroxyguanine released by iron-dead cells activated, thereby triggering ferroptosis (Hirschenberger et al., 2023). It indicates that STING1 regulates cellular ferroptosis in a variety of mechanisms. In addition, endoplasmic reticulum stress-associated calcium ion efflux triggers the endocytosis-associated complex Endosomal Sorting and Transport Complex-III (ESCRT-III) to accumulate in the plasma membrane to prevent membrane damage during ferroptosis (Lin et al., 2023). This suggests that during ferroptosis, the ER is crucial for preserving the functionality of the cell membrane and averting damage to the membrane. Overall, the ER role in ferroptosis comprises multiple aspects of protein synthesis and processing, lipid peroxidation, regulation of specific proteins, and maintenance of membrane integrity, suggesting that the ER has significance to ferroptosis.

## 5 Metabolic pathway and signaling pathways related to ferroptosis

### 5.1 GPX4-GSH metabolic pathway

The formation of glutathione (GSH) and the activity of glutathione peroxidase 4 (GPX4) are important components of this pathway. Cysteine and glutamate transfer throughout the cell membrane and GSH synthesis relies on the Xc-antioxidant system, comprised of the SLC7A11 and SLC3A2 bis-subunits (Chu et al., 2019). When cystine is taken up by the xc-system, GSH reductase reduces it to cysteine. The thioredoxin reductase 1 (TRXR1)/thioredoxin 1 (TRX1) system also maintains a reduced pool of GSH without GSH reductase (Prigge et al., 2017). Cysteine is a nonessential amino acid, and under certain conditions, methionine can be converted via the transsulfuration pathway to homocysteine, cystathionine, and ultimately cysteine (Dixon et al., 2015; Zou et al., 2020; Yan et al., 2021). Suchprocess is subject to the cysteine-tRNA synthetase I being negatively regulated (Hayano et al., 2016). Thus, inhibition of the transsulfuration pathway is expected to increase sensitivity to ferroptosis. The cytoplasmic enzymes glutathione synthetase (GSS) and GCL mediate the conversion of glycine, glutamate, and cysteine into GSH. Additionally, GPX4 contributes to detoxification by accelerating the transformation of H_2_O_2_ and hydroperoxides into water or ethanol (Bela et al., 2015), relying on the effectiveness of glutathione as a cofactor. In addition to its major cofactor GSH, GPX4 demonstrates versatility by employing various low-molecular thiols, including protein thiols. Selenoprotein GPX4's active site contains selenocysteine (Sec). Additionally, the synthesis of selenoproteins, such as GPX4, is regulated by the mevalonate (MVA) pathway. The biological activity of GPX4 and the synthesis of GSH collaborate to inhibit ferroptosis in cells at a molecular level (Soupene and Kuypers, 2008). Erastin, Due to their specific inhibition of the Xc-system, sorafenib and sulfasalazine (SAS) suppress GPX4 directly by depleting GSH, while RSL3 effectively blocks GPX4 function (Yang et al., 2014). Inhibiting intracellular cysteine and GSH levels affects GPX4 function since cysteine is the rate-limiting precursor for GSH formation and GSH is a major antioxidant in mammalian tissues, thereby making cells more susceptible to ferroptosis (Soupene and Kuypers, 2008).

It has been shown that there are interactions between various organelles and the GPX4-GSH metabolic pathway. For the intracellular interaction between lysosomes for cells to remain in a state of redox equilibrium and to avoid ferroptosis, the GPX4-GSH metabolism process is mandatory. The primary intracellular digesting and recycling organelles, lysosomes are in charge of breaking down and recycling a range of macromolecules, such as degraded fatty acids, and nucleic acids. In the GPX4-GSH metabolic pathway, lysosomes may regulate GPX4 activity by degrading damaged GPX4 or affecting its upstream molecules (Deng et al., 2023). In addition, iron ions released by lysosomes may indirectly affect GPX4 activity because to scavenge lipid peroxides, GPX4 requires GSH as a reductant, and iron ions may affect the synthesis or utilization of GSH. The GPX4-GSH metabolic pathway is also capable of affecting lysosomes. Ferroptosis can be avoided by scavenging lipid peroxides that are activated with iron ions, owing to the important antioxidant enzyme known as GPX4 (Xue et al., 2023). GPX4 requires GSH as a reductant to fulfill its function. In the GPX4-GSH metabolic pathway, GPX4 activity may directly affect lysosomal stability and function. For example, when GPX4 activity is reduced, the intracellular lipid peroxidation level is elevated, which may result in damage to the lysosomal membrane, thereby affecting the normal function of lysosomes. The maintenance of lipid peroxidation levels, protein synthesis, and cellular redox equilibrium depends on the interactions between the ER and the GPX4-GSH metabolic pathway. In addition to being linked to the formation of lipids and metabolism, the ER is the primary site for protein production and modification in cells. GPX4 can scavenge lipid peroxides catalyzed by iron ions, thereby preventing ferroptosis. GPX4 requires GSH as a reductant to perform its function (Jia et al., 2024b). The ER supports the activity of GPX4 by synthesizing and supplying antioxidants, such as GSH, thereby helping cells to resist peroxidation of lipid and ferroptosis. Moreover, the production and function of GPX4 are controlled by the ER and modulates its antioxidant capacity by affecting the post-translational modification or stability of GPX4. The GPX4-GSH metabolic pathway positively affects ER function by scavenging lipid peroxides and maintaining cellular redox homeostasis (Chen et al., 2017). Lipid peroxidation damages the ER membrane and the proteins therein, leading to ER stress and dysfunction. By scavenging lipid peroxides, GPX4 can reduce the damage to the ER and maintain its normal function. In cell metabolism and antioxidant defense systems, there is a significant interaction between the GPX4-GSH metabolic process and mitochondria. The important internal organelles known as mitochondria are in control of generating energy and engaging in a variety of metabolic activities. Within GPX4-GSH metabolic pathway, mitochondria provide reduced GSH to GPX4 as a reductant essential to its antioxidant activity. GSH, through the action of GPX4, can scavenge phospholipid hydroperoxides catalyzed by ferric ions, thereby preventing ferroptosis. Therefore, the mitochondrial function has a direct impact on the antioxidant capacity of the GPX4-GSH metabolic pathway, which also affects the mitochondria. by scavenging phospholipid hydroperoxides catalyzed by ferric ions, the GPX4-GSH metabolic pathway prevents lipid peroxidation damage to the mitochondrial membrane, thus maintaining the mitochondrial functional and structural integrity (Asperti et al., 2021). Furthermore, the GPX4-GSH metabolic pathway can lessen OS-induced mitochondrial destruction by scavenging ROS in mitochondria (Yuan et al., 2021). Consequently, the GPX4-GSH pathway's activation is required for the mitochondria to function normally. In summary, there is an interaction between various intracellular organelles and the GPX4-GSH metabolic pathway. This interaction is important for maintaining cellular redox homeostasis and preventing lipid peroxidation and ferroptosis. Thus, thorough research on the relationship between organelles and the GPX4-GSH metabolic pathway could yield important insights into the management or avoidance of ferroptosis-related disorders.

### 5.2 FSP1-CoQ/DHODH-CoQ/GCH1-BH4/Nrf2-Keap1 signaling pathway

In the last few years, three GPX4-independent signaling pathways have been identified to inhibit ferroptosis: Ferroptosis Suppressor Protein 1 (FSP1)/CoQ10, Dihydroorotic acid dehydrogenase (DHODH), and GTP Cyclic Hydrolyserase 1 (GCH1)/Tetrahydrobiopterin (BH4). Bersuker et al. (2019) and Doll et al. (2019) discovered the CoQ10-FSP1 route, an innovative ferroptosis inhibition pathway. At cell membrane, FSP1 behaves as an oxidoreductase to promote NADPH oxidase-catalyzed CoQ10 to generate CoQ10H_2_ (Shimada et al., 2016). This pathway synergizes with GSH-GPX4 to inhibit ferroptosis.

Two more GPX4-independent systems that prevent lipid peroxidation and ferroptosis are reported in 2020 and 2021. In 2020, it was found that GCH1 regulates the levels of PLs through two PUFAs tails to produce the lipophilic antioxidant BH4, defends cells against ferroptosis (Kraft et al., 2020). The first defense mechanism against DHODH-mediated mitochondrial ferroptosis was initially shown by Boyi's group in 2021. To avoid ferroptosis in the inner membrane of the mitochondria, DHODH lowers CoQ to CoQH2. This reaction occurs in tandem with mitochondrial GPX4 (Mao et al., 2021). Ferroptosis was unable to affect cells with high levels of GCH1 or DHODH, and was more probable to affect cells with low expression. Antioxidant proteins are produced under the control of Nrf2, which protects cells from oxidative damage. Kelch-like ECH-associated protein 1 (Keap1) controls its activity. When Keap1 attaches to Nrf2 in the cytoplasm, Nrf2 becomes ubiquitinated and is then degraded by the proteasome (Song and Long, 2020). Nrf2 is translocated to the nucleus and released from the Keap1 binding site in response to oxidative damage. To stimulate the transcription of protective antioxidant genes, Nrf2 can interact with antioxidant response elements (AREs) in the nucleus (Zhang, 2006). Autophagy has been a regulatory mechanism for these activities. By deactivating Keap1, the multipurpose protein p62, which is found in the cell and acts as an autophagy receptor, stimulates Nrf2 (Komatsu et al., 2010). The p62-Keap1-Nrf2 antioxidant signaling pathway is responsible for the inability of hepatocellular carcinoma (HCC) cells to enter ferroptosis, as revealed by Tang's group in 2016 (Sun et al., 2016). The p62-Keap1-NRF2 pathway was shown to be significant in defending HCC from ferroptosis. Multiple genes involved in the breakdown of iron and ROS, such as quinone oxidoreductase-1 (NQO1), heme oxygenase-1 (HO1), and ferritin heavy chain 1 (FTH1), are stimulated to produce that protective effect (Sun et al., 2016). In addition, the genes SLC7A11 and GSS, which encode GSH synthesizing proteins, are also considered Nrf2 targeted genes (Sun et al., 2016). These findings imply that Nrf2 is crucial for ferroptosis resistance.

It has been shown that there are interactions between various organelles and the signaling path between Nrf2-Keap1. Lysosomes and the Nrf2-Keap1 signaling pathway interact in a complicated and important way, and is important for regulating cellular redox balance and shielding cells from xenobiotic invasion and oxidative damage. By attaching to and increasing the breakdown of Keap1, a significant Nrf2 inhibitor that keeps Nrf2 levels low, lysosomes modify the processes of the Nrf2-Keap1 pathway (Amorim et al., 2022). Lysosomes can degrade Keap1 through their internal acidic environment and abundance of hydrolytic enzymes, thereby relieving the inhibitory effect on Nrf2. When lysosomal function is impaired or subjected to certain stimuli, the degradation of Keap1 may be blocked, leading to the accumulation and Nrf2 stimulation. Lysosomes are the subject of the Nrf2-Keap1 activation pathway. By controlling the levels of antioxidant proteins and detoxify enzymes, the Nrf2-Keap1 signaling pathway preserves cellular redox equilibrium. They protect the lysosomal membrane from oxidative damage and maintain lysosomal stability and function. In addition, activation of Nrf2 promotes autophagy (Meeran et al., 2021). Therefore, the Nrf2-Keap1 signaling pathway can affect lysosomal function and activity by regulating autophagy. The interaction between ER and the Nrf2-Keap1 signaling pathway is important for maintaining cellular homeostasis, coping with oxidative stress, and regulating protein synthesis and degradation. ER is not only a major site of protein synthesis and modification but also takes part in several signal transduction pathways (Cong et al., 2021). In the Nrf2-Keap1 signaling pathway, ER may regulate the activity of Nrf2 by affecting the modification and stability of Keap1. For example, certain enzymes in the ER may modify Keap1 and affect its ability to bind to Nrf2, thereby regulating the stability and transcriptional activity of Nrf2. In addition, ER stress may also affect the activity of the Nrf2-Keap1 signaling pathway, which enhances cellular antioxidant capacity by inducing the degradation of Keap1 or promoting the nuclear translocation of Nrf2. On the other hand, the Nrf2-Keap1 signaling pathway maintains cellular redox homeostasis by regulating the expression of antioxidant proteins and detoxifying enzymes. These antioxidant proteins and detoxifying enzymes protect the ER from damage by oxidative stress and maintain its normal function (Park et al., 2016) The Nrf2-Keap1 signaling system and mitochondria interact to a great extent in cell metabolism, redox homeostasis, and antioxidant defense. One of the primary contributors of intracellular ROS is superoxide, which is formed in the mitochondrial oxidative respiratory chain. The Nrf2-Keap1 pathway's activity may be affected by certain ROS. The thiol moiety of Keap1 may be oxidized in the presence of high ROS levels, resulting in a conformational shift that releases Nrf2 (Masuda et al., 2024). To preserve cell redox equilibrium, the emitted Nrf2 subsequently reaches the nucleus and starts the transcription of antioxidant genes. Furthermore, modulation of the Nrf2-Keap1 process may raise the synthesis of certain enzymes that detoxify and antioxidant proteins that can scavenge ROS and prevent oxidative stress-induced mitochondrial injury. In addition, some antioxidant proteins can be directly localized in mitochondria, such as mitochondrial thioredoxin and mitochondrial GPX, which are directly scavenging ROS, preserve mitochondrial structure and function (Sun et al., 2024). In summary, there is an interaction between various intracellular organelles and the Nrf2-Keap1 pathway. This interaction is important in preventing lipid peroxidation and ferroptosis, among others.

### 5.3 cGAS-STING signaling pathway

The cGAS-STING [(cGAMP) synthase (cGAS)-interferon gene-stimulating factor (STING)] signaling pathway is a mediator of the inflammatory response under stress, infection, and tissue injury. Cell-microbe and host-derived double-stranded DNA (dsDNA) can be sensed and regulated for defense against extracellular or intracellular infection (Hopfner and Hornung, 2020; Decout et al., 2021). Then, the production of the second messenger (cGAMP) is catalyzed by cGAS (Cheng et al., 2020). Subsequently, STING bound to cGAMP oligomerizes at the endoplasmic reticulum membrane and then translocates from the ER to the Golgi compartment, mediating tank-binding kinase 1 (TBK1) autophosphorylation (Cheng et al., 2020). As a result, TBK1 phosphorylates STING, which then attracts interferon (IFN) regulatory factor 3 (IRF3) to continue phosphorylation (Cheng et al., 2020). IFN-stimulated genes (ISGs) and type I IFN transcription are mediated by phosphorylated IRF3 dimers, which are moved to the nuclei and engaged in an array of pathological events (Jiang et al., 2014a,b; Negishi et al., 2018; Cheng et al., 2020).

Studies have revealed an association between the cGAS-STING process and ferroptosis. Various studies attested to STING's role in the ferroptosis mechanism. According to Li et al., mitochondrial oxidative damage was brought on by erastin, an established inducer of ferroptosis. Moreover, mitochondrial STING expedited the mitochondrial fusion that depends on the mitofusin gene (MFN1/2), which leads to ferroptosis and peroxidation of lipids (Li et al., 2021a). Meanwhile, by decreasing the vulnerability of cells to ferroptosis through the inhibition of STING or MFN1/2, erasin's tumor-suppressive effects were reduced (Li et al., 2021a). Furthermore, IRF3 is involved in ferroptosis and peroxidation of lipids as an alternative target of STING. Docosahexaenoic acid (DHA) decreased ALOX activity, raised IRF3 expression, aided SLC7A11 transcription, and halted ferroptosis in a mouse model of cardiac hypertrophy carried on by angiotensin II (Shi et al., 2022). There could be an effect of ferroptosis on the cGAS-STING regulation pathway. Jia et al. (2020) found that the stimulation of the cGAS-STING signaling mechanism was hampered by an increased cell peroxidation of lipids brought on by GPX4 knockdown. Moreover, ferroptosis in mice brought on by a high-iron diet or a GPX4 loss is shown to stimulate kras-mediated pancreatic tumor formation, based on research by Dai et al. (2020). Integrating results suggests that ferroptosis and lipid peroxidation are related to the active cGAS-STING regulation mechanism, and that iron prolapse's detrimental effects can be lessened by concentrating on this mechanism.

**Figure 2:**
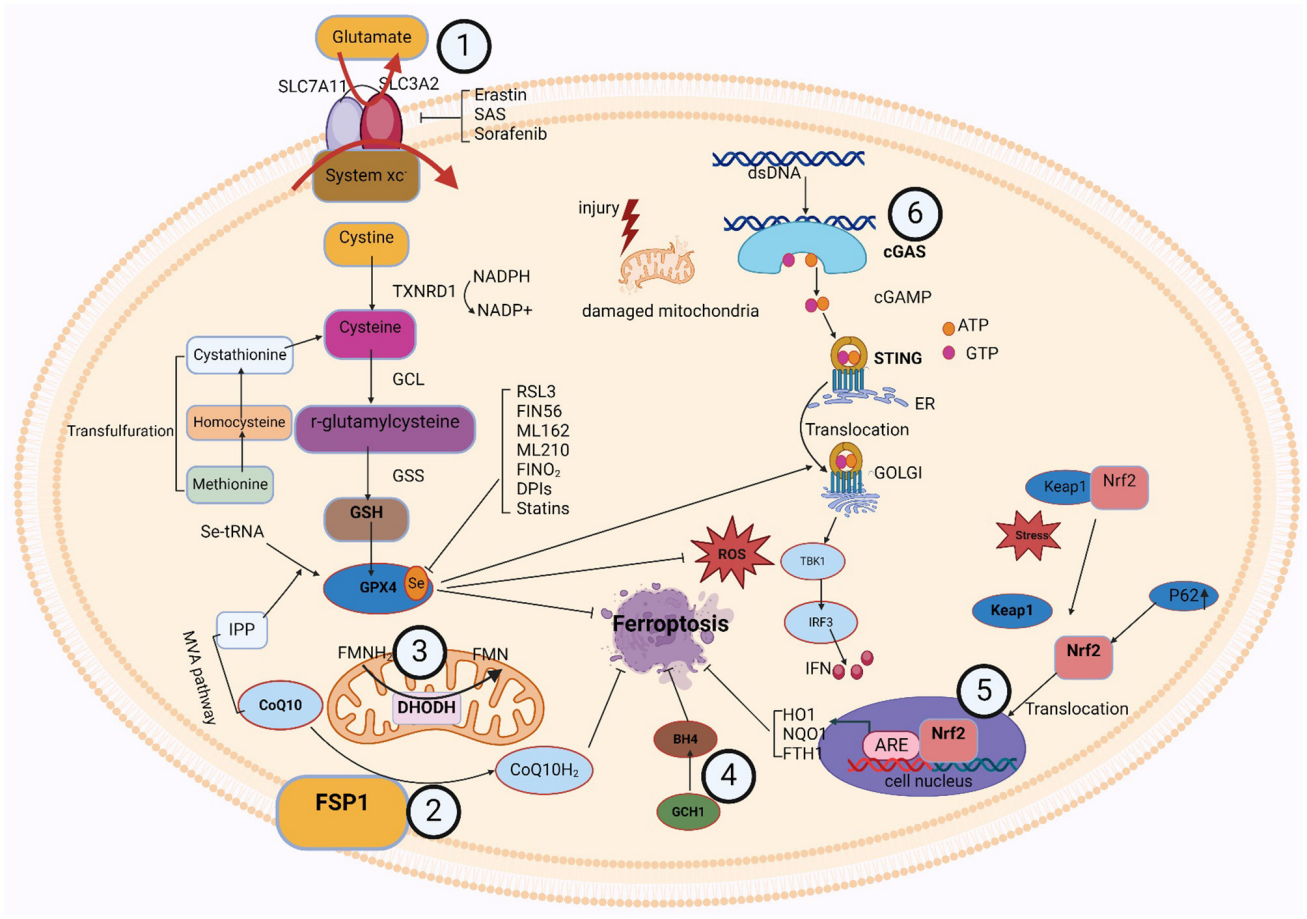
The signaling pathways associated with ferroptosis.

It was discovered that numerous organelles engage with the cGAS-STING signaling cascade. The association between lysosomes and the cGAS-STING signaling cascade has gradually gained more attention in recent years. The implications of this association extend to cellular immunity and signaling. In addition to breaking down a variety of macromolecules, lysosomes are intracellular recycling and digesting organs that may also affect the activity of the cGAS-STING cascade (Gaidt et al., 2017). First, lysosomes may negatively regulate the activity of the pathway by degrading cGAS or STING proteins. Second, iron ions or other molecules released by lysosomes may directly affect the enzymatic activity of cGAS or the phosphorylation state of STING, thereby modulating the activity of the pathway. However, lysosomes are also affected by the cGAS-STING signaling mechanism. When the cGAS-STING pathway detects intrinsic DNA damage or DNA viruses, it initiates a cascade of signaling events that trigger the synthesis of inflammatory mediators and the recruitment of lymphocytes. This activation state may indirectly affect lysosomal function. For example, activated immune cells may enhance bactericidal or anti-tumor capabilities by releasing lysosomal enzymes (Wu et al., 2024). Furthermore, by controlling the expression of specific transcription variables, the cGAS-STING signaling network may have an impact on lysosomal formation and function the interaction between ER and the cGAS-STING signaling pathway plays an important role in cellular immunity and antiviral responses. ER is not only a major site of protein synthesis and modification but also participates in a variety of signal transduction processes. The ER offers an area for the cGAS to react in the cGAS-STING signaling process, allowing the cGAS to identify and engage DNA in the cell's cytoplasm before activating and synthesizing the second messenger, cGAMP. Through its unique membrane structure, cGAS-STING also gives STING an anchor site, allowing STING to locate and operate on the ER membrane (Thomsen et al., 2024). cGAS-STING signaling pathway is also capable of acting on the ER. The second messenger, cGAMP, is produced by cGAS upon recognition and binding to DNA in the cytoplasm, activating STING. From the ER, active STING travels to the Golgi apparatus, where it recruits and activates signaling molecules downstream, including IRF3 and TBK1. Type I interferon is produced and secreted as a result of these communication pathways, and this initiates an antiviral immune response (Gong et al., 2024). The structure and function of ER may be affected in some way during this process, such as induction of ER stress and alteration of protein synthesis. The key component of cellular immunity and the antiviral response is played by the association between mitochondria and the cGAS-STING regulating cascade. As significant intracellular organelles, mitochondria not solely serve as the main site for generating energy but also take part in several different cell signaling endeavors. Mitochondria may activate cGAS in the cGAS-STING signaling circuit by producing ROS. The amount of ROS rises in mitochondrial infection or damage. These ROS can trigger cGAS, which may then attach and identify DNA in the cytoplasm, activating and synthesizing cGAMP, and cGAS will attach to DNA in the cell cytoplasm after recognizing it. Upon identifying and attaching itself to cytoplasmic DNA, cGAS produces another messenger, cGAMP, which then triggers STING to initiate a sequence of transmitted signals cascades (Jiménez-Loygorri et al., 2024). This series of signaling processes leads to the synthesis and secretion of type I interferon, which in turn triggers an antiviral immune response. During this process, mitochondria may be affected in certain ways, such as changes in mitochondrial function and fluctuations in ROS levels. Moreover, the mitochondrial autophagy process may be impacted by the initiation of the cGAS-STING method, which maintains cellular homeostasis by removing damaged or excess mitochondria (Zhou et al., 2024). In summary, there is an interaction between various intracellular organelles and the Nrf2-Keap1 pathway. This interaction is important in preventing lipid peroxidation and ferroptosis, among others. Figure 2 shows the signaling pathways associated with ferroptosis.

## 6 Pathologic relationship between ferroptosis and neurodegenerative diseases

The hallmark of neurodegenerative diseases is the gradual death of neurons and are brought on by an accumulation of oligomers and aggregates of misfolded proteins (Menzies et al., 2017; Djajadikerta et al., 2020). Ferroptosis has been linked to several mechanisms that occur in neurological disorders, including peroxidation of lipids, GSH depletion, and brain depositing iron (Reichert et al., 2020). Moreover, ferroptosis in motor dementia was linked to reduced functioning of the GSH and GPX4 networks (Johnson et al., 2012; Dias et al., 2013; Blesa et al., 2015; Gu et al., 2015). For healthy neural development and mental ability, iron is important. With age, higher amounts of iron are observed in the caudate nucleus, pallidum, shell nucleus, substantia nigra, and other basal ganglia and cortex regions. Certain areas of the body accumulate iron, which is associated with neurological disorders. Iron overload in the brain can lead to GPX4 deficiency and lipid peroxidation in neurons and glial cells. Research has identified an association between motor nerve deterioration and increased iron (Chen et al., 2015; Reichert et al., 2020). Deposits of iron in specific brain regions can hasten the death of neurons and glial cells in neurological disorders. The inflammation of the brain is marked by immune cell infiltration into areas with small lesions, and this process is frequently linked with brain inflammation (Urrutia et al., 2021). Under a microscope, the primary sites of brain iron formation are dystrophic microglia invaded by astrocytes and iron-containing macrophages. Recent work shows that increased cytoplasmic levels of iron can cause microglia to polarize toward an inflammatory M1 phenotype (Kroner et al., 2014). It was proposed that the neuroinflammatory reaction can be affected by the polarization of microglia (Cherry et al., 2014). Iron increases into deep gray matter and cortical astrocytes and microglia as individuals age, but it stays constant in oligodendrocytes (Connor et al., 1990). Usually, iron accumulates in oligodendrocytes, where it is primarily stored as ferritin and transferrin. These levels of iron remain unchanged with aging. Oligodendrocytes are the primary source of iron and irons-binding proteins including ferritin and TRF. Cell damage can be triggered immediately by iron deposits in neurons (Williams et al., 2012). The potential medicines to inhibit the development of AD and PD have been proposed, including ferritin 1 and iron chelators such as Deferoxamine (DFO), M30, and lipoic acid. These substances may work as protectors by making hypoxia-inducible factor 1α (HIF1α) in the brain more stable. The tissue of the brain is rich in PUFA lipids, including arachidonic acid (AA) and docosahexaenoic acid (DHA), which also has an increased lipid content, fewer antioxidant enzymes, and higher oxidative metabolism (Olmez and Ozyurt, 2012; Larrieu and Layé, 2018). In the brain's tissue, peroxidation of lipids becomes accessible. Multiple studies have found that increased intracellular liberated radical oxidative damage linked to iron metabolism is part of the cause of neurological diseases (Stockwell et al., 2017; Mao et al., 2020). Because ferroptosis exerts a role in the pathomechanism of neurodegenerative diseases, targeting it could provide therapeutic opportunities for their treatment.

### 6.1 Ferroptosis and AD

Alzheimer's disease (AD) is a common neurodegenerative condition in older adults that focuses primarily on the hippocampal and cerebral cortex. The AD pathogenesis is typified by the development of Tau protein-containing neurofibrillary knots and senile plaques, which are composed of Aβ polypeptides (Serrano-Pozo et al., 2011; 2022). Genetic alterations in AD include changes in the genes encoding amyloid precursor protein (APP), apolipoprotein E (ApoE), presenilin 1 (PSEN1), and presenilin 2 (PSEN2) (Scheltens et al., 2021). APP cleaves to form amyloid beta (Aβ), while hyperphosphorylation leads to the separation and aggregation of Tau proteins from microtubules, creating tangles in nerve fibers of nerve cells. Physiologically, APP enters the non-amyloid pathway via alpha-secretase and gamma-secretase cleavage. Amyloidosis arises from the aberrant cleavage of APP by β- and γ-secretases, releasing neurotoxic fragments such as Aβ40 and Aβ42, which are capable of aggregating and subsequently developing plaques that contribute to responsible for AD (Yan and Zhang, 2019). Mutations in PSEN1 and PSEN2, part of the catalytic protease compound that precisely cleaves APP and other proteins, and are linked to AD with early manifestations (Walter, 2015). The plasma membrane of neurons is rich in PUFAs and is susceptible to free radical attack and peroxidation of unsaturated carbon-carbon bonds. Pathogenesis of the nervous system is related to ROS (Yang and Stockwell, 2008).

Recent evidence suggests that Ferroptosis may play an integral part in AD development. In the brain, iron is a biological element that is vital to several significant processes. The synthesis of neurotransmitters, myelin formation, oxidative phosphorylation, and transport of oxygen are some of these processes (Ward et al., 2014). Recent research exists to support the direct induction of ferroptosis by perturbations in iron regulation, both *in vivo* and *in vitro* (Wang et al., 2017). Iron-storing proteins and Cp were shown at higher levels in AD patients than in healthy people, based on Ashraf et al. However, it has been shown that AD sufferers express DMT1 and FPN1 at reduced levels (Ashraf et al., 2020). Age-related iron formation was shown by Raven et al. (2013) to be a factor in damage to tissues, cognitive loss, and clinical AD manifestations. Neuronal toxic levels decreased substantially when Aβ had been treated by using the iron chelator DFO (Rottkamp et al., 2001). In rats, amyloid-beta deposition and tau excess phosphorylation have been exacerbated by a high-iron meal; whereas, iron chelators successfully decreased amyloid beta deposition and tau excess phosphorylation (Sripetchwandee et al., 2014). In the APP/PS1 mutant mice species, Svobodová et al. (2019) the formation of amyloid plaques in the brain cortex corresponds to the accumulation of ferritin and liberated iron. Additionally, It is already known that iron accumulates in the body and leads to the abnormal formation of Aβ and neurofibrillary tangles (NFTs), resulting in Aβ plaque misfolding (Yan and Zhang, 2019). Extensive experimental studies showed that the beta-amyloid interacts with iron, leading to Aβ to clump together and become cytotoxic (Bousejra-ElGarah et al., 2011; Lermyte et al., 2019). In contrast, iron has an eight-fold higher binding affinity for Aβ than TRF, thereby interfering with iron homeostasis (Jiang et al., 2009). The aggregation of proteins associated with tau is a significant hallmark of AD. Iron induces and regulates tau protein phosphorylation (Nikseresht et al., 2019; Yan and Zhang, 2019). Wan et al. (2019) found that as compared to control group, neurons exposed to ferrous iron displayed 110% higher phosphorylated tau protein at S396. Amyloid precursor protein (APP) was recently identified by Tsatsanis et al. (2020) to regulate the cellular-surface expression of membrane-bound iron transporters, hence improving neural iron efflux. Tau deficiency renders APP-mediated iron export impure, causing an increase of iron inside cells. Furthermore, the advancement of AD and cognitive impairment has been associated with increased brain iron concentrations. Currently, magnetic resonance imaging (MRI) techniques are highly sensitive, and MRIs show iron accumulation in the nucleus of the pallidum, caudate and chiasmatic nuclei, and also in specific cortical region (van Duijn et al., 2017). In the neocortex and deep gray matter of the brain, AD patients had increased levels of iron than healthy controls, as shown in a subsequent study. Moreover, cognitive loss in AD sufferers was linked to changes in temporal lobe iron content with time (Damulina et al., 2020). When Aβ and tau interact, glial cells become hyperactive, a range of pro-inflammatory mediators are released, directing further inflammatory compounds toward the point of damage, leading to neural inflammation (Dhapola et al., 2021). It was shown that microglial iron overload reduces the secretion of insulin-degrading enzymes, which degrade Aβ and subsequently cause neurotoxicity by extracellular Aβ accumulation (Liu et al., 2022). The aberrant activation of microglia is caused by disruptions in brain iron equilibrium and neural ferroptosis, which contribute to enhanced neural inflammation. The release of inflammatory mediators by these improperly activated microglia exacerbates iron homeostasis disruptions and promotes neuronal inflammation (Wang M. et al., 2023). More studies has identified new ferroptosis symptoms in AD individuals, including elevated peroxidation of lipids and decreased systemic Xc-activity. Modifications in glutathione levels impact redox homeostasis and are linked with a higher risk of ferroptosis in AD individuals (Pocernich and Butterfield, 2012; Hambright et al., 2017). In an experimental model, iron accelerated neural cellular death in the context of low GSH levels by reducing GCL activation (Maher, 2018). The glial cells' synthesis of GPX4 and GSH has been diminished in mild hypoxia (Makarov et al., 2006). Lipid peroxidation products formed during iron death lead to increased metabolites downstream of the AD brain, including 4-HNE, MDA, and acrolein. It was shown that elevated 4-HNE content in the brains of people with AD causes damaging carbonylation of proteins reactions (Lovell et al., 1997; Jaganjac et al., 2020; Shin et al., 2020). Further research has demonstrated that 4-HNE binds to Aβ polypeptides to form covalent complexes, promoting covalent cross-linking of Aβ polypeptides and protofibril formation (Siegel et al., 2007). 4-HNE disrupts Na^+^/ca2^+^ pumps as well as glucose and glutamate transporters by modifying cell membranes, leading to ionic and energetic disturbances and neuronal cell death (Keller et al., 1997; Mark et al., 1997). The results show that the neuropathologic hallmark of AD is also influenced by the release of peroxidation of lipids byproducts. They observed a decline in GPX4 expression in AD model mice and sufferers (Ansari and Scheff, 2010), indicating that substantial hippocampus nerve damage and cognitive decline may occur in GPX4 knockout mice (Yoo et al., 2010). Using the GPX4BIK0 mouse, an animal model of selective GPX4 loss in forebrain neurons, Hambright et al. showed that ferroptosis could be a significant AD disease (Hambright et al., 2017). They observed AD-like cognitive deficits in GPX 4 BIKO mice. Additionally, GPX 4 BIKO mice showed a substantial decrease in hippocampal neuronal degeneration and spatial-temporal memory retention, and ferroptosis is considered to be a significant AD factor. In clinical practice, two regularly utilized iron chelators are deferiprone (DFP) and DFO (Part et al., 2015). DFO can be up to 50% clinically effective in treating AD (Farr and Xiong, 2021). It has been shown that the application of the iron chelator DFO in the APP/PS1 double transgenic model activates the expression of heat shock protein B1 and inhibits the transfer of iron ions by the TFR (Fine et al., 2015). Many other iron prolapse inhibitors have also been associated with AD. Chalcone 14a-c inhibits Aβ aggregation and protects neuronal cells from Aβ aggregation-induced toxicity and erastin- and RSL3-induced ferroptosis in human neuroblastoma (Nemeth et al., 2004). The presence of hydroxyl groups in chalcone derivatives inhibited toxicity and ferroptosis caused by Aβ plaque aggregation. In an animal model, Ates et al. showed that CMS121-induced fatty acid synthase (FASN) inhibition reduced the peroxidation of lipids. In comparison to untreated mice of the wild-type, CMS121 therapy decreased 15-AL0X levels in the hippocampal regions. By preventing tau-induced excess iron and increasing the expression of GPX 4, α-lipoic acid prevents ferroptosis and reduces lipid peroxidation and brain damage in AD (Hambright et al., 2017). Deuterium-enhanced PUFAs reduced cortical and hippocampal lipid peroxidation, improved cognitive performance in an aldehyde dehydrogenase 2 (ALDH2)^_/_^mouse model, and reduced Aβ in APP/PS1 transgenic mice. Mice's cognitive abilities and neurodegeneration were enhanced by vitamin E or the ferroptosis inhibitor lipoprostane 1 administration, whereas a vitamin E-deficient diet simultaneously worsened hippocampal neurodegeneration and behavioral dysfunction (Hambright et al., 2017). It was shown that the memory of Aβ-induced AD mice was improved by the administration of fer-1 and Lip-1, and the effect of Lip-1 on memory was more significant (Bao et al., 2021). Baicalein reduces oxidative stress, inhibits ALOXs, and serves as a neuroprotective and anti-inflammatory agent. Mice fed baicalein-APP/PS1 exhibited superior cognitive test outcomes, reduced levels of Aβ and p-tau, and lowered beta-site amyloid precursor cleaving enzyme-1 (BACE1) activity (Xie et al., 2016; Li et al., 2019). Epigallocatechin gallate (EGCG) has antioxidant, anti-inflammatory, and neuroprotective effects. When EGCG was administered to AD mice, it showed that EGCG's protective properties were attained by lowering APP and Aβ expression in the hippocampal regions. Early clinical studies confirmed the neuroprotective and anti-inflammatory properties of EGCG against brain swelling and injury to neurons (Plascencia-Villa and Perry, 2021). Hepcidin ameliorated cognitive decline and partially reduced Aβ plaque formation in the cortex and hippocampus of APP/PS1 mice. In the HT22 cell line treated with erastin and RSL3, sterubin compounds maintained GSH levels, suggesting an anti-ferroptosis effect (Fischer et al., 2019). 5-Lipoxygenase (5-LOX) is an important regulator of lipid metabolism pathways, and 5-LOX inhibitors inhibit ferroptosis in mouse hippocampal neurons and exert neuroprotective effects. Iron depressor 1 reduces oligodendrocyte mortality by inhibiting PUFA oxidation in membrane lipids (Gascón et al., 2016).

### 6.2 Ferroptosis and PD

The most prominent chronic neurodegenerative disorder has emerged as Parkinson's disease. Clinically, PD patients may have dementia risk in addition to non-motor and motor impairments (Fearnley and Lees, 1991). The etiology of PD remains elusive. New genomics research has revealed that PD's autosomal dominant family rarely occurs by variants in the alpha-synuclein (α-syn) gene, while autosomal recessive familial PD is caused by alterations in the parkin gene. The principal pathogenic features of PD comprise the distinct degradation of neuronal dopaminergic function and intracellular buildup of α-synuclein. Resident brain microglia-mediated neuroinflammation may be an important mediator of dopaminergic neuron loss (Ko et al., 2021). Neuroinflammation is caused by the change and proliferation of activated microglia, which produce activated microglia and release several pro-inflammatory substances (Tang and Le, 2016). A significant increase in M1 marker gene activity and a decrease in M2 marker gene regulation are triggered by α-syn over-expression. As a result, mice have reduced motor development and dopamine neuron degeneration (Rodriguez-Perez et al., 2018; Ko et al., 2021). Thus, the brain inflammatory process closely correlates with the pathophysiology of PD.

Recent studies have shown that Ferroptosis was correlated to PD development (Do Van et al., 2016). Recently, other significant variables of PD etiology have been identified, such as iron development, an increase in oxidative stress, and peroxidation of lipid degradation. The basal ganglia, a region of the brain associated with elevated iron levels in the elderly and related to PD, can accumulate elevated levels of iron (Rouault, 2013; Damulina et al., 2020; Depierreux et al., 2021). MRI showed increased iron content in the dense portion of the substantia nigra and the caudal part of the nucleus accumbens. Exposure to iron and ROS resulted in the emergence of a neurodegenerative M1 phenotype in microglia, which was marked by increased levels of iNOS and higher levels of inflammatory cytokines, including IL6, IL1β, and tumor necrosis factor (TNF) (Ko et al., 2021). The subsequent study revealed that iron overload impacts the nigrostriatal system overall, with the substantia nigra (SN) serving as the primary target beginning in the initial phases of PD. The PD progression can be tracked using potential MRI biomarkers in the substantia nigra striata. Such markers may reveal early stages of striatal atrophy, new phases of iron overload, and changes in the morphology of the striatum (Hopes et al., 2016). In advanced PD phase, elevated iron levels may also cause neuroinflammation (Fenton's reaction) via high ROS levels (Shi et al., 2019). Mutations in iron-regulated proteins lead to abnormal iron homeostasis and are linked to PD pathology (Guiney et al., 2017). The SN dense neurons that produce dopamine and mitochondria both contain TFR2 protein. The inherited difference in TFR1 and TFR2 corresponds with dopaminergic neuron iron homeostasis (Rhodes et al., 2014). In PD patients and animal models, higher DMT1 levels and decreased Cp ferrous oxidase activity were noted; both outcomes potentially resulted in elevated iron contents (Salazar et al., 2008; Ayton et al., 2013). In PD patients, increased DMT1 expression causes iron deposits, oxidative damage, and neuronal death. DMT1 mutations protect rodents from the PD-induced neurotoxins 1- methyl-4-phenyl-1,2,3,6-tetrahydropyridine (MPTP) and 6-hydroxydopamine (6-OHDA) (Salazar et al., 2008). The emergence of PD has been attributed to genetic variations exhibiting Cp (Ayton et al., 2013). Moreover, iron flow is disturbed by the loss of APP. After 21 days of therapy, a decrease in nigral APP was seen in the 1-methyl-4-phenyl-1,2,3,6-tetrahydropyridine (MPTP) mice model, which corresponded with higher iron contents. Comparably, the substantia nigra (SN) of the APP knockout mouse model (APP-/-) exhibited high levels of iron (32%). Additionally, Ayton et al. hypothesized that neurodegeneration in PD patients may be facilitated by minimized levels of APP. Furthermore, in α-Syn-rich inclusion bodies, upregulation of α-Syn resulted in increased cellular iron levels and a redistribution of iron from the cell's cytoplasm to the perinuclear area. Moreover, giving the iron chelator deferiprone (DFP) to 3-month-old APP-/- mice decreased the loss of SN neurons, suggesting that an iron-dependent process plays a role in the initiation of neuronal loss in APP-/- animals (Ayton et al., 2015).

Aberrant cerebral deposits of iron are hypothesized to represent one of the pathogenetic routes of PD, particularly in the more severe stages of the condition [An et al., 2018, Lewy bodies (LB(s)] are formed by iron by the oxidative damage mechanism (Takahashi et al., 2007). A study found that the concentration of iron in the thick portion of the SN in PD individuals may rise gradually throughout the disease (181). In the middle and final phases of the disease's course, this study found higher levels of iron deposition (Li et al., 2018). In the SN area, glial cells and neural cells accumulate excessive iron, which is often correlated with the severity of the condition (Hirsch et al., 1991). Individuals with leucine-rich repeat kinase 2 (LRRK2) and Parkin polymorphisms have been found to have elevated iron deposition in the SN. Decreased internal GSH levels may contribute to the oxidative damage observed in PD, which is consistent with one of the potential causes of ferroptosis (Costa et al., 2023). PD has also been associated with impaired GPX4 activity. Bellinger et al. observed that the brains of PD patients had substantially lower levels of glutathione peroxidase 4 (GPX4) than the brains of controls (those without PD). Additionally, it was found that in PD brains, the cellular density of survived SN neurons was linked with a rise in GPX4 immune response. This implies that the loss of cells in PD is linked to GPX4 expression (Bellinger et al., 2011). Through the reduction of OH·-, RO2·-, and peroxynitrite, GSH shields neural cells from damage caused by cellular oxidative stress. Consequently, it serves as an indispensable antioxidant in brain tissue (Dringen et al., 2000). Thus, lowered glutathione content causes mitochondrial malfunction, which causes cellular stress and damages the mitochondria (Lee et al., 2009). PD nigral tissue autopsy analysis shows decreased GSH contents (Fitzmaurice et al., 2003). In a small phase, GSH exhibits promising I/II phase outcomes in PD treatment studies (Mischley et al., 2015). The findings from an additional preliminary study demonstrated that N-acetylcysteine therapy improved motor coordination (Monti et al., 2016). In conclusion, ferroptosis may play a part in the pathophysiology of PD due to the increased lipid peroxidation, iron, and decreased glutathione levels found in the brains of PD individuals and disease animals. Current PD therapies are intended to increase dopamine (DA) production and thus relieving symptoms (Mahoney-Sánchez et al., 2021). Commonly used therapeutic agents include the dopamine precursor levodopa (L-DOPA), dopamine agonists, and inhibitors of dopamine metabolism (Connolly and Lang, 2014). In recent years, targeting ferroptosis has emerged as one of the ways to alleviate the symptoms of PD due to its increasing importance in its pathogenesis. It was demonstrated that iron chelators, including DFO, improve motor manifestations by lowering damage from oxidative stress and raising dopaminergic activity in neurons (Do Van et al., 2016). According to studies on brain scans, DFP improves motion issues, modifies ferritin in the CSF fluid, and lowers cerebral iron concentrations (Martin-Bastida et al., 2017). DFO, ferritin 1, and D-PUFAs reduced oligomerization-induced neuronal cell death. In addition, D-PUFAs prevented α -Syn-induced neuronal cell loss (Angelova et al., 2015). Copper(II) diacetyl-bis(N4-methylthiosemicarbazone) [CuII(atsm)]-mediated activation of Nrf-2-related anti-oxidant enzymes was shown to promote dopamine metabolic rate, protect SN cells from peroxidation of lipids, and improve motor and cognitive abilities. Such results are confirmed by experiments conducted using both *in vivo* and *in vitro* PD models (Hung et al., 2012; Southon et al., 2020). Calcium pool-operated Ca^2+^ influx (SOCE) repopulates the endoplasmic reticulum after Ca^2+^ release. In the 1-methyl-4-phenylpyridinium (MPP+)-induced PD model, cells are protected against ferroptosis by the pharmaceutical or genetic inhibition of SOCE-related proteins (Maher et al., 2018). Furthermore, astrocytes can prevent iron overload in neurons by taking up and storing iron (Codazzi et al., 2015). Glutathione s-transferase Mu2 and other antioxidants are supplied by astrocytes to protect neurons from oxidative damage. Bacitracin A1 and chloroquine, two ferritin phagocytosis inhibitors, decreased ferritin degradation and ferroptosis in 6-OHDA-treated PC12 cells, suggesting that FTH1 overexpression might have neuroprotective properties. More studies identified a link between ferritin phagocytosis and ferroptosis in the 6-OHDA model of PD, indicating that FTH1 may serve as a novel target for future drug discovery (Plascencia-Villa and Perry, 2021). Therefore, targeting iron prolapse is also one of the treatments to improve PD symptoms.

### 6.3 Ferroptosis and HD

Huntington's disease (HD) is a familial, delayed-onset neurological disorder with an age of onset that ranges between 30 to 50 years. Rapid, involuntary movements and cognitive impairment are its main characteristics. Amplification of CAG repeat sequences in Huntington's protein (HTT) ultimately leads to death. The key trigger and pathological feature of HD is the ease with which mutant Htt (mHtt) cleaves and aggregates into toxic macromolecules, leading to neuronal degeneration and cell death. To be precise, mHtt's N-terminus is cut and cleaved to produce monomers or tiny oligomeric fragments with abnormal forms, namely β-sheet configurations. These damaging fragments of cytoplasm may affect the proteasome, chaperonins, and neuronal autophagy systems that break down dysfunctional proteins. Additionally, anomalies in the mitochondria such as decreased ATP and higher ROS may be mediated by these perilous fragments (Ayala-Peña, 2013). Iron accumulation and abnormal glutamate and GSH levels were also seen in HD (Skouta et al., 2014; Agrawal et al., 2018).

Ferroptosis may contribute to the genesis of HD, given that patients with HD have limited plasma GSH levels and lowered GPX4 expression in their erythrocytes. Furthermore, HD was linked to elevated peroxidation of lipids, iron accumulation, and Glutathione depletion, all indicators of ferroptosis (Klepac et al., 2007; Chen et al., 2013). Research revealed after 3-nitro propionic acid (3-NP) was administered to animals with HD, an inhibition in GSH and GSH-s transferase (GST) was observed in the striatum, cerebral cortex, and hippocampal regions (Kumar et al., 2010). However, in this HD model, supplementation with cysteine and Cysteine reduced GSH and lessened neuronal harm triggered by 3-NP (Mao et al., 2006). A frequent observation in HD is iron formation, which is a major cause of iron demise. HD patients have elevated basal ganglia striatal iron and ferritin levels (Bartzokis and Tishler, 2000; Bartzokis et al., 2004, 2007). The levels of iron in the pallidum, shell nucleus, and mature nuclei are higher in HD individuals' MRI scans (Bartzokis et al., 1999). Using inductively coupled plasma mass spectrometry (ICC MS), another study confirmed that the cortex and striatum of R6/2 HD mice contained larger quantities of iron (Fox et al., 2007). Although the precise processes causing ferroptosis in brain tissue are still unknown (Magtanong and Dixon, 2018). Elevated iron may be caused by HTT mutations. In mHtt transgenic mice, Berggren et al. concluded that the inhibition of APP was responsible for the accumulating the iron in brain tissue (Berggren et al., 2017). Concurrently, mHTT enhanced and further increased iron homeostatic factors (such as ferritin, TRF, IRP1, and TFR) in the striatum and cortex of HD transgenic mice's brains, indicating that mHTT may cause an excess of iron in HD by increasing IRP1 (Niu et al., 2018). A further study revealed that microglia are mostly responsible for the abnormalities in the breakdown of iron in HD patients and are crucial for HD development (Simmons et al., 2007). High iron further aggravates neurodegenerative damage in HD (Mi et al., 2019). In HD, significant peroxidation of lipids was also noted. Increased plasma peroxidation of lipids has been identified in hemodialysis patients (Klepac et al., 2007). Cortical striatal brain slices showed significant peroxidation of lipids (Skouta et al., 2014), cerebrospinal fluid (CSF) (Reddy and Shirendeb, 2012) and striatal neurons in multiple HD animal models (Lee et al., 2011). The increased oxidative stress brought on by mHTT raises the amount of ROS in cells (Wyttenbach et al., 2002). Furthermore, it emerged that mHtt inclusion bodies co-localized with striatal neurons that had elevated lipid peroxidation levels (Lee et al., 2011), implies that peroxidation of lipids and iron mortality is a factor in mHtt and HD. Moreover, HD patients had greater levels of the lipid peroxidation variables f2-isoprostane, 4-hydroxytryptophan, and MDA (Mariani et al., 2005; Lee et al., 2011).

There are currently no particular drugs available for the HD treatment. The primary objective of treatment choices is to reduce the patient's symptoms, which include weakness, choreiform actions, and developing dementia (Jimenez-Sanchez et al., 2017). Since ferroptosis may be associated with the HD process, treatments targeting iron prolapse may have beneficial effects. Iron chelation therapy with DFO, DFP and DFX has been successful in reducing excessive iron accumulation, decreasing peroxidation of lipid and improving activity of mitochondrial cells (Grolez et al., 2015). After reversal of iron elevation with the iron chelator DFO, R6/2 HD mice showed gradual improvement in rotarod endurance and significant reduction of the ventricle on the treated side, suggesting a neuroprotective effect (Chen et al., 2013). Furthermore, in the HD brain slice model, a method that decreased the peroxidation of lipids with ferritin 1 minimized cellular death (Skouta et al., 2014). Similarly, glucose-dependent insulin stimulation of the polypeptide (GiP) receptor D-Ala 2GiP and deuterium-enhanced linoleic acid ameliorated neurobehavioral deficits and attenuated cognitive deficits in HD patients by reducing lipid peroxidation (Hatami et al., 2018; Verma et al., 2018). Heat shock protein 27 (HSP27) reduces ROS in expressing mHtt cells and protects cells from oxidative stress. Activation of the antioxidant gene Nrf2 increased the expression of target genes such as HO-1, thereby enhancing antioxidant defense (Dinkova-Kostova et al., 2018). Thus, certain approaches targeting Nrf2 signaling may delay the onset of neurodegenerative diseases, slow progression, and improve symptoms (Stack et al., 2010).

### 6.4 Ferroptosis and ALS

A severe and long-lasting neurodegenerative disease named amyotrophic lateral sclerosis (ALS) primarily affects motor nerve cells in the central nervous system (CNS) (Kiernan et al., 2011). About 10% of ALS cases are inherited and due to genetic changes, even though most cases are irregular and the etiology is unidentified (Veyrat-Durebex et al., 2014). Initially identified as the gene associated with modifications in ALS, variations in SOD1 are currently responsible for 20% of cases of ALS with familial and 3% of cases of sporadic ALS (Jeong et al., 2009). The function of ferroptosis in ALS has been outlined in a variety of investigations.

Various studies showed aberrant accumulation of iron in ALS-affected mice, and also in individuals with inherited and sporadic ALS (Veyrat-Durebex et al., 2014; Gajowiak et al., 2016; Moreau et al., 2018). Iron accumulation was found in the spinal cord of an animal model of ALS and the CNS [including the motor cortex, SN, pallidum, erythrocyte, and chiasma nuclei of ALS patients (Acosta-Cabronero et al., 2018; Moreau et al., 2018)]. Also, various proteins such as DMT1, TFR1, membrane FPN, and plasma Cp were increased in the spinal cord of ALS animals (Jeong et al., 2009). The indicators of oxidative damage and peroxidation of lipids, comprising MDA, 4-HNE, protein carbonylation, and oxygen membrane phospholipids, were found in experimental animals and samples from hereditary and random ALS cases (Valentine, 2002; Perluigi et al., 2005). Oxidation-related stress markers are more common in the spinal cord and various bodily fluids of ALS patients (e.g., plasma and cerebrospinal fluid) and raised NOX2 enzyme activity. A critical role for NOX2 has also been reported in studies using the SOD1 animal model and *in vitro* models (e.g., organotypic spinal sections). Importantly, motor nerve deterioration and immobility result from GPX4 loss in neurons that prevent ferroptosis (Chen et al., 2015). On the other hand, GPX4 upregulation delayed the progression of the neurological disorder in mutant SOD1G93A ALS mice (Chen et al., 2021). However, synthesis of GSH disruption and GPX4 deletion has been shown in ALS mutant mice (Wang et al., 2022). In addition, GPX 4 neuron-induced knockout (GPX 4 NIKO) mice showed motor neuron degeneration, ALS-like paralytic symptoms and spinal motor neuron death (Evans et al., 2022). GPX4 overexpression in ALS reduces the cytotoxicity of SOD1, delays ALS onset, improves motor neuron function, and prolongs survival (Chen et al., 2021).

Multiple studies have demonstrated the treatment of ALS by inhibiting iron death in motor neurons. DFP has recently been shown to improve mean lifespan in a mouse model of ALS (SOD1G86R) and has also been effective in an insufficient group study of patients with ALS and (Moreau et al., 2018). Iron chelators such as DFO and SIH reduce pathological iron accumulation in ALS, improve spinal motor neuron survival, and restore motor function (Jeong et al., 2009; Moreau et al., 2018; Masaldan et al., 2019). In addition, studies on the ferroptosis inhibitor [CuII(ATSM)] have shown that it prevents lipid peroxidation and ferroptosis in an ALS cell model (Southon et al., 2020). Clinically authorized for the treatment of ALS, daravone is a scavenger of free radicals that was found to reduce motor neuron damage in ALS patients and block iron death and cystine deficit in systemic Xc -/GPX 4 inhibition (Spasić et al., 2020; Al-Chalabi et al., 2021). Although there are fewer reports on the treatment of ALS, its future does deserve attention. Table 3 shows the role of different ferroptosis drugs in neurodegenerative diseases.

**Table 3:** The significance of different ferroptosis drugs in neurodegenerative diseases.

**Disease**	**Drug**	**Therapeutic mechanism**	**References**
AD	Alpha-lipoic acid (LA)	Block tau-induced iron overload, lipid peroxidation, and inflammation	Zhang Y. H. et al., 2018; Ji et al., 2022
	CoQ10	Inhibition of Aβ plaque formation	Sultana et al., 2013
	Fer-1	Aβ, inhibits ROS levels and upregulates Nrf 2 and GPX 4 to reduce angiotensin II-induced astrocyte inflammation and ferroptosis	Gwon et al., 2010
	Lip-1	Reduction in Aβ	Friedmann Angeli et al., 2014
	4-O-methylhonokiol	Inhibition of β-secretase expression, lipid peroxidation and enhancement of GPX4 activity	Di Meco et al., 2017; Vitalakumar et al., 2021
	PD146176	Reduction of Aβ and iron deposition levels, attenuation of tau protein neuropathy, inhibition of 12/15 - LOX-mediated PUFA oxidation, and attenuation of cognitive decline in an AD mouse model	
	hydroxylated chalcones	Inhibits Aβ aggregation and prevents RSL4/erastin induced ferroptosis	Cong et al., 2019
	DFO	Improves iron excretion and reduces the amount of iron in the body deposited in various organs	Ji et al., 2022
	Clioquinol	Decreasing tau oligomerization and decreasing tau dimension in brain tissue by reducing the levels of APP and β and γ-secreting enzymes	
	Vitamin E	Reduce lipid peroxidation and weaken iron form	
	Hepcidin	Iron absorption and discharge from neurons decreases when FPN1, TFR1, and DMT1 are suppressed.	Du et al., 2015; Ji et al., 2022
	Selenic compound	In patients with AD, overexpression of GPX 4 may prevent ferroptosis and enhance cognitive function.	Gwon et al., 2010; Ji et al., 2022
	Insamgobonhwan (GBH)	It can prevent cellular death and peroxidation of lipids *in vitro* and prevent Aβ from impairing brain function *in vivo*.	Yang et al., 2022
M30	Decreased Tau phosphorylation and activated HIF-1α signaling pathway	Han et al., 2020
PD	DFP	Decreased oxidative stress and increased DA activity	Ji et al., 2022
	Clioquinol	Chelated iron, antioxidant	
	D-PUFA	Prevents loss of neuronal cells induced by α-Syn	Angelova et al., 2015
HD	DFO	Enhance iron excretion and decrease accumulation of iron in organs	Ji et al., 2022
	Fer-1	Inhibits oxidative lipid damage	
	HSP27	Decreased ROS expression in mHtt cells and protected cells from OS	Wyttenbach et al., 2002
	Nrf2	Increased expression of target genes such as HO-1, thereby enhancing antioxidant defense	Dinkova-Kostova et al., 2018
ALS	Deferiprone	Chelate iron	Moreau et al., 2018
	Edaravone	Inhibition of cystine deficiency and systemic Xc -/GPX 4 inhibition, scavenging free radicals	Southon et al., 2020; Spasić et al., 2020; Al-Chalabi et al., 2021
	CuII(ATSM)	Prevents lipid peroxidation and ferroptosis	Nikseresht et al., 2020
	DFO	Iron chelate, improve spinal motor neuron survival and restore motor function	Jeong et al., 2009; Keuters et al., 2021

## 7 Conclusions and prospects

Ferroptosis is an iron-dependent form of cell death that has been defined and refined by many researchers in recent years. However, the mechanism of ferroptosis remains controversial. Initially, how can ferroptosis result from lipid peroxidation? Molecular dynamics simulations and other computational techniques can help study the characteristics of the membrane during ferroptosis. It is unclear, how peroxidation of lipids products including MDA and 4-HNE function in ferroptosis. Additionally, it's unknown where the peroxidation of lipids is located within cells. The default location is at the plasma membrane of the cell, and lipid peroxidation is unlikely to occur at other subcellular sites: mitochondria, endoplasmic reticulum, and lysosomes. The susceptibility of different organelles to lipid peroxidation may vary depending on iron stores, GSH levels, LOX expression, lipid composition, and GPX4 localization. This challenge may be solved by identifying the various organelles' chemical composition and how they encourage or prevent ferroptosis and peroxidation of lipids. Finally, for diseases caused by ROS excess (e.g., neurodegenerative diseases), clinical studies have demonstrated that antioxidants are essentially ineffectual, most likely because of their inadequate, late, and very nonspecific impact. Therefore, specific inhibition of ROS-generating enzymes (e.g., NADPH oxidase) is a more promising approach to clinical efficacy. However, the majority of NOX inhibitors remain in the early phases of study., and there is a lack of NOX inhibitors that can specifically act on NOX proteins, selectively act on different NOX isoforms and have favorable pharmacological effects. In addition, the existing NOX inhibitors are mainly obtained by high-throughput screening. Although high-throughput screening can rapidly screen a variety of compounds, the specificity of the screened compounds for NOX proteins and their selectivity for different NOX subtypes cannot be guaranteed. Therefore, a complete and reliable screening system is necessary for the future discovery of selective NOX inhibitors. Understanding these intriguing issues could lead to fresh perspectives and innovative therapies for neurological diseases. There is a dearth of clearly defined therapeutic methods due to the complexity of the etiology of neurological disorders and the unknown direct source of caused neuronal death. The physiological aspects of ferroptosis have come to light as possible causes of neurological disorders, providing fresh perspectives on the processes underlying neuronal death. Ferroptosis is a potential therapeutic target since it contributes to the development and course of certain neurodegenerative disorders. With therapeutically indicated ferroptosis targets including DFO, iron chelators are widely used in neurodegenerative diseases. When treating AD, DFO has shown up to 50% therapeutic effectiveness. However, side effects such as appetite suppression and weight loss make clinical application challenging. In addition, DFO has difficulty crossing the blood-brain barrier (BBB), which can now be addressed by intranasal administration of DFO nanoparticles (Crapper McLachlan et al., 1991; Ward et al., 1995; Shachar et al., 2004; Rassu et al., 2015). In addition, DFP crosses the blood-brain barrier and is safer compared to DFO (Hider and Hoffbrand, 2018). Initial trials of DFP in PD patients have shown that it can reduce iron deposition in the SN and potentially prevent or slow disease progression. A follow-up multicenter phase II trial is currently underway (Devos et al., 2014). Furthermore, although iron chelators strongly remove intracellular iron in mammalian models, protect neurons from oxidative damage, and have demonstrated efficacy in treating systemic iron deposition; however, there is a significant clinical risk of medical iron deficiency and the ensuing anemia with these drugs. In contrast, several studies have shown that modest iron chelation regimens that avoid changes in systemic iron levels offer a new therapeutic modality for neuroprotection, particularly in long-term treatment regimens (Devos et al., 2014; Keuters et al., 2021). Furthermore, research is being conducted on tiny-molecular ferroptosis antagonists treating AD, PD, and ALS (Homma et al., 2019; Southon et al., 2020; Keuters et al., 2021). Clinical studies have identified the effectiveness of iron chelators and antioxidants as effective alternatives for reducing ferroptosis in neurological conditions. However, their reduced efficacy in human studies highlights the necessity of looking into signaling molecules associated with different pathways that induce ferroptosis. Despite these obstacles, an increasing amount of research highlights the crucial part ferroptosis plays in several neurological disorders. Further research in this arena may offer encouraging results and breakthrough therapeutics for people with neurological diseases.

## Author contributions

LW: Supervision, Writing—original draft. XW: Supervision, Writing—review & editing. TZ: Investigation, Writing—review & editing. XF: Writing—review & editing, Methodology. BL: Writing—review & editing, Methodology, Formal analysis. FW: Writing—review & editing, Methodology, Formal analysis. YX: Writing—review & editing, Methodology, Formal analysis. WZ: Writing—review & editing, Methodology, Formal analysis.

## Abbreviations

AD: Alzheimer's disease
PD: Parkinson's disease
ALS: amyotrophic lateral sclerosis
HD: Huntington's disease
ROS: Reactive oxygen species
TRF: transferrin
TfR1: transferrin receptor 1
NCOA4: nuclear receptor coactivator 4
HSP90: heat-shock protein 90
IL-1β: interleukin 1β
IL-18: interleukin 18
GZMA proteins: human granzyme family members
GSDMB: Gasdermin structural domain protein family member
IFN-γ: gamma interferon
SLC7A11: solute carrier family 7 member 11
GSH: glutathione
PUFAs: polyunsaturated fatty acids
ALOX: arachidonic acid lipoxygenase
AA: arachidonic acid
AdA: adrenoic acid
PE: phosphatidylethanolamine
ACSL4: acetyl coenzyme a synthetase long-chain family 4
LPCAT3: lysophosphatidylcholine acyltransferase 3
CPY450: cytochrome P450 oxidoreductase
hephaestin: ferrous oxidase
TFR1: transferrin receptor 1
STEAP3: prostate hexagonal transmembrane epithelial antigen 3
DMT1: divalent metal transporter protein 1
NCOA4: nuclear receptor coactivator 4
FPN1: iron transporter protein 1
CP: Ceruloplasmin
RSL3: GSH peroxidase 4 inhibitor
HO-1: heme oxygenase-1
NRF2: nuclear factor erythrocyte 2-related factor 2
PLOOH: phospholipid hydroperoxides
MDA: malondialdehyde
4-HNE: 4-hydroxynonenal
GPX4: glutathione peroxidase 4
PCBP1: chaperonin poly(rC)-binding protein 1
ACSL4: acyl coenzyme a synthase long-chain family member 4
LPCAT3: lysophosphatidylcholine acyltransferase 3
PTGS2: Prostaglandin peroxidase synthase 2
PPAR: peroxisome proliferator-activated receptor
OS: Oxidative stress
NADPH: nicotinamide adenine dinucleotide phosphate
NOX: NADPH oxidase
XO: xanthine oxidase
DPP4: dipeptidyl peptidase 4
VDAC: voltage-dependent anion channel
PLs: phospholipids
CoQ10: coenzyme Q10
GCL: Glutamate cysteine ligase
BSO: L-buthionine sulfoximine
TBH: t-butyl hydroperoxide
Fer-1: Ferrostatin-1
Lip-1: Liproxstatin-1
SOD1: Superoxide dismutase 1
ETC: electron transport chain
MGST1: Microsomal glutathione S-transferase 1
STING1: Stimulator Of Interferon Response CGAMP Interactor 1
ESCRT-III: Endosomal Sorting and Transport Complex-III
ER: endoplasmic reticulum
GSS: glutathione synthetase
Sec: selenocysteine
MVA: mevalonate
SAS: Sulfasalazine
DHODH: Dihydroorotic acid dehydrogenase
GCH1: GTP Cyclic Hydrolyserase 1
BH4: Tetrahydrobiopterin
Keap1: kelch-like ech-related protein1
AREs: antioxidant response elements
HCC: hepatocellular carcinoma
cGAS-STING: (cGAMP) synthase-interferon gene-stimulating factor
dsDNA: double-stranded DNA
TBK1: tank-binding kinase1
IFN: interferon
IRF3: interferon regulatory factor3
ISGs: IFN-stimulated genes
MFN: mitofusin gene
DHA: docosahexaenoic acid
DFO: Deferoxamine
HIF1α: Hypoxia-inducible factor 1α
APP: amyloid precursor protein
APP: apolipoprotein E
PSEN1: progerin1
PSEN2: progerin2
Aβ: Amyloid beta
NFTs: neurofibrillary tangles
MRI: magnetic resonance imaging
DFP: deferiprone
BACE1: beta-site amyloid precursor cleaving enzyme-1
TNF: tumor necrosis factor
MPTP: 1-methyl-4-phenyl-1,2,3,6-tetrahydropyridine
DA: dopamine
6-OHDA: 6-hydroxydopamine
APP-/-: APP knockout mouse model
SN: substantia nigra
LB(s): Lewy bodies
D-PUFAs: Deuterated polyunsaturated fatty acids
DA: 3-hydroxytyramine
Cu(II)ATSM: a positron emission tomography developer sensitive to hypoxia
MPP+: 1-methyl-4-phenylpyridinium
HTT: Huntington's protein
mHtt: mutant Htt
3-NP: 3-nitropropionic acid
GST: glutathione S-transferase
HSP27: Heat shock protein 27
CNS: central nervous system
SOD1: Superoxide dismutase1
GPX 4 NIKO: GPX 4 neuron induced knockout.
